# Forkhead box P3 gene polymorphisms predispose to type 2 diabetes and diabetic nephropathy in the Han Chinese populations: a genetic-association and gender-based evaluation study

**DOI:** 10.1186/s41065-023-00264-1

**Published:** 2023-01-31

**Authors:** Xiaorong Wang, Zejing Liu, Shangdi Zhang, Yinfeng Yang, Xue Wu, Xinyue Liu

**Affiliations:** 1 Department of Pharmacogenomics Laboratory Center, Lanzhou University Second Hospital, Lanzhou, 730030 Gansu China; 2 Department of Clinical Laboratory Center, Lanzhou University Second Hospital, Lanzhou, 730030 Gansu China; 3 Lanzhou University Second Hospital, Lanzhou, 730030 Gansu China

**Keywords:** FOXP3, T regulator cells, Gender-based evaluation, Single nucleotide polymorphism, Type 2 diabetes mellitus, Diabetic nephropathy

## Abstract

**Background:**

Functional mutations or polymorphisms affecting forkhead box P3 (*FOXP3)* can lead to their abnormal FOXP3 gene expression and/or defective Treg cells generation, thus resulting in autoimmune disease and inflammatory disorders. FOXP3 also plays a key role in Type 2 diabetes mellitus (T2DM) and its complications, because the disease usually involves chronic low-grade inflammatory disorders and is associated with long-term immune system imbalance. This study aimed to investigate the association between FOXP3 polymorphisms and the susceptibility to T2DM and type 2 diabetes nephropathy (T2DN) within the Han Chinese populations.

**Methods:**

Polymorphisms in rs3761548C/A and rs2294021C/T were examined in 400 patients (which include an equal number of T2DM and T2DN groups) and 200 healthy controls using PCR-HRM and sequence analysis.

**Results:**

The genotype and allelic frequencies of the two single nucleotide polymorphisms (SNPs) were significantly different in T2DM and the progression of diabetes developing to T2DN. The further gender-based evaluation showed that in female subjects, rs3761548C/A was associated with an approximately 3-fold higher threat for T2DM and 4.5-fold for T2DN, while there was no noticeable association with rs2294021C/T; in males, the promoter polymorphism showed an increased predisposition of 5.4-fold and 3.4-fold predisposition to T2DM and T2DN, respectively, while rs2294021 polymorphism could impart a nearly 2-fold risk of developing T2DN. An additional analysis of combined genotypes (rs3761548 C/A-rs2294021C/T) revealed that CC-CC and CC-CT can be considered protective combinations in the predisposition of males with diabetes towards T2DN, while AA-CC and AA-TT have the opposite effect.

**Conclusions:**

This study demonstrated the possible involvement of individual and combined genetic associations of rs3761548C/A and rs2294021C/T polymorphisms with the susceptibility to diabetes and diabetic nephropathy in the Han Chinese population, as well as gender bias.

## Background

As one of the most serious and highly prevalent chronic global diseases in the twenty-first century, diabetes has caused countless life-threatening and disabling and complications. According to the Diabetes Atlas of the International Diabetes Federation, China has the largest number of diabetics with estimates of over 140 million in 2021, and reaching over 174 million by 2045 [34]. More than 1 in 10 adults now have diabetes mellitus globally, 90% of whom have type 2 diabetes mellitus (T2DM). Approximately 20 ~ 40% of these diabetic patients may develop to type 2 diabetic nephropathy (T2DN), which is an extremely common and progressive complication of T2DM in China [17, 22].

As is well-established, T2DM is a metabolic disorder caused by multiple genetic loci and environmental factors that lead to impaired insulin secretion, glucose intolerance and hyperglycemia [8, 17]. It has also been demonstrated that T2DM is a chronic and low-grade inflammatory disease associated with long-term immune system imbalance, metabolic syndrome, or overnutrition in obesity [32]. Many of the earlier studies linking inflammation and diabetes have focused on the innate immune function, represented by macrophages and neutrophils, as well as other innate cells such as mast cells and dendritic cells (DCs) that have been well established to contribute to T2DM pathogenesis [9, 31, 40]. However, recent studies have pointed out that the adaptive immune system, especially Treg cells (CD4 ^+^ CD25 ^+^ regulatory T cells), a subpopulation of CD4^+^ T cells, also exerts important effects in the regulation of chronic inflammation and further participate in the pathogenesis of abnormal energy metabolism, such as T2DM [38, 43]. Treg cells play a key role in maintaining immune tolerance and immune homeostasis, they protect inflamed tissues from harmful immune responses by inducing self-tolerance, down-regulating the function of the effector cells, and curbing the inflammatory processes that promote the occurrence and progression of T2DN. In addition to this, microalbuminuria, macroalbuminuria, and urinary albumin-creatinine ratio (UACR) were negatively related to the Tregs levels in T2DM patients, indicating that the imbalance of Tregs may contribute to immune activation and inflammatory progression in T2DN [30, 41].

Forkhead box P3 (*FOXP3*), a member of the forkhead–winged-helix family of transcription factors, has emerged as an expert regulator of the pathways involved in the growth, differentiation and function of Tregs [13]. The *FOXP3* gene, located on chromosome Xp11.23, measures 21 kb, and comprises of a 5′-untranslated region and 11 protein-encoding exons [27]. Mutations in *FOXP3* can lead to an autoimmune lymphoproliferative syndrome that involves immune dysregulation, polyendocrinopathy, enteropathy X-linked syndrome (IPEX) in humans, and an analogous X-linked pathology in the scurfy (sf) mutant mice [4, 7]. Polymorphisms in various regions of *FOXP3* gene including the promoter, intron and exon region, may change its role functionally or quantitatively, and thus give rise to the dysfunction of Tregs, resulting in some chronic inflammation and certain autoimmune diseases [3, 16]. More interestingly, sex-specific differences have been increasingly recognized and reported in FOXP3 genetic mutations involved in diseases, such as ulcerative colitis (UC) [39], rheumatoid arthritis (RA) [19] and Multiple Sclerosis (MS) [10].

Based on the above background, we hypothesized that the varying associations of FOXP3 variants are likely attributed to the genetic variations in the different populations. Therefore this study aimed to understand the relationship between the functional polymorphisms (rs3761548C/A and rs2294021C/T) of *FOXP3* gene and the genetic susceptibility to T2DM and T2DN as well as the gender influence among the Han Chinese population.

## Materials and methods

### Sample collection and ethics

All the study subjects and related data shown were recruited at the Second Affiliated Hospital of Lanzhou University during 2019–2020. A total of 600 individuals were enrolled in the present study from the Han Chinese population, which included an equal number of T2DM patients, T2DN patients, and healthy controls (HC). The inclusion criteria for the three groups were as follows: (i) 200 T2DM patients diagnosed with a urinary albumin excretion < 30 mg/day, negative for dipstick urinary proteins, and long-standing diabetes for at least 5 years with no history of hypertension before the development of diabetes; (ii) 200 T2DN patients diagnosed with urine albumin excretion > 300 mg/day without any clinical evidence of other kidney diseases, or infectious conditions; and (iii) 200 healthy controls were selected from the physical examination centers (HC group). The diagnosis of the subjects was based on commonly accepted clinical and laboratory criteria from Lanzhou University Second Hospital in Gansu Province, Northwest China. Individuals with a history of cancer, SLE, RA, MS, connective tissue disease, renal disease, cancer, chronic wasting disease, primary cardiovascular disease or liver or lung disease were excluded from the study.

### Clinical parameters detection

The demographic data of the patients, such as age, sex, body mass index (BMI), age at onset of the disease, and duration of T2DM are presented in Table 1. The related biochemical indicators, including the triglyceride (TG), total cholesterol (TC), low-density lipoprotein cholesterol (LDL-C), high-density lipoprotein cholesterol (HDL-C), fasting and post-lunch blood sugars (FBS and PLBS), as well as the urea and serum creatinine levels, were analyzed using routine clinical methods. The glycosylated hemoglobin (HbA1c) levels were estimated using a Nycocard reader (AXIS SHIELD, Norway).

**Table 1:** Demographic profile and clinical characteristics of HC, T2DM and T2DN

Characteristics	HC(*n* = 200)	T2DM(*n* = 200)	T2DN(*n* = 200)	t-test *p*-value
Males / Females (%)	48.5/51.5	57.5/42.5	72/28	–
Age (years)	57.57 ± 10.95	61.64 ± 9.80	60.05 ± 10.82	0.135
Disease duration	–	13.16 ± 6.35	13.41 ± 5.78	0.707
Age at Onset of T2DM (yrs.)	–	48.48 ± 9.53	46.64 ± 9.96	0.069
BMI (Kg/m^2^)	22.44 ± 0.5	23.85 ± 2.65	24.30 ± 3.32	0.136
Hba1c (%)	–	12.14 ± 3.59	9.23 ± 2.14	0.405
FBS mg/dl	–	10.12 ± 3.77	10.47 ± 4.86	0.416
PLBS mg/dl	–	12.25 ± 4.57	12.67 ± 4.79	0.372
Total cholesterol (mg/dl)	3.81 ± 0.96	4.35 ± 1.14	4.11 ± 1.33	0.064
Triglyceride (mg/dl)	1.29 ± 0.55	1.91 ± 1.24	2.10 ± 2.16	0.285
HDL cholesterol (mg/dl)	1.03 ± 0.26	1.15 ± 0.29	1.07 ± 0.31	**0.018**
LDL cholesterol (md/dl)	2.57 ± 0.74	2.85 ± 0.87	2.68 ± 0.88	**0.047**
TC/HDL ratio	3.84 ± 0.96	3.92 ± 1.04	4.01 ± 1.39	0.495
TG/HDL ratio	1.37 ± 0.73	1.88 ± 1.57	2.31 ± 2.95	0.070
Urea(mg/dl)	6.25 ± 2.43	6.79 ± 3.23	9.45 ± 6.63	**< 0.001**
Creatinine (mg/dl)	57.88 ± 19.04	69.00 ± 55.38	126.88 ± 168.85	**< 0.001**
Urea/Creatinine ratio	0.11 ± 0.04	0.11 ± 0.03	0.10 ± 0.03	**0.010**

Test of significance (t-test) was analyzed between T2DM and T2DN. *HC* Healthy control, *T2DM* Type 2 Diabetes Mellitus, *T2DN* Type 2 Diabetic Nephropathy, *BMI* Body Mass Index, *HbA1c* Hemoglobin A1c, *FBS* Fasting Blood Sugar, *PLBS* Post Lunch Blood Sugar, *TC* Total Cholesterol, *TG* Triglycerides, *HDL* High-Density Lipoproteins, *LDL* Low-Density Lipoproteins

### DNA extraction and primer design

Genomic DNA was extracted from EDTA-containing whole blood using the TIANamp Genomic DNA Kit (TIANGEN, China), according to the procedure described by the manufacturer. The DNA purity and concentration were determined by measuring the absorbance at 260 nm and 280 nm, using a spectrophotometric analyzer (DENOVIX DS-11, USA), which was further confirmed by 1% agarose gel electrophoresis.

The primers were synthesized by the TSINGKE Biotech (Xian, China) as follows: 5′ CTCTAGGTGGGACCCTGGTT3′ (forward) and 5′ GATCGTGGATCGTCCAACCT 3′ (reverse) for rs3761548C/A and 5′ TCCTTCTGAAGCTCCTTCGT3′ (forward), 5′ CAATCCATCCCAGTCACCCC 3′ (reverse) for rs2294021C/T.

### PCR-HRM techniques and genotyping

A Rotor-Gene Q PCR Amplification Instrument (Qiagen Corp.) with a 36-well rotor was used for both the PCR and high-resolution melting (HRM) steps. The reaction consisted of 10.0 μl Forget-me-Not Evagreen qPCR Master Mix (Biotium, USA), 0.5 μl of 10 μM forward and reverse primer, 0.5 μl 40X template buffer, 1 μl of the DNA sample with 7.5 μl PCR-grade distilled water adjusted to a total volume of 20 μl. The 154 bp amplicon of rs3761548 was run according to the following conditions: initial denaturation at 95 °C for 5 min followed by 40 cycles of denaturation at 94 °C for 35 s, thereafter annealed at 57.2 °C for 35 s with a reading of the fluorescence, subsequently, high resolution melting was performed from 80 °C to 90 °C at a ramp rate of 0.2 °C/s. The 158 bp amplicon of rs2294021 was run according to the following conditions: initial denaturation at 95 °C for 5 min followed by 40 cycles of denaturation at 95 °C for 35 s, thereafter annealed at 55.6 °C for 40 s with a reading of the fluorescence; subsequently, melting was performed from 80 °C to 90 °C at a ramp rate of 0.2 °C/s. Sequencing analysis was performed on the samples using the PCR products obtained after the melting analysis, and the results from the HRM analysis were confirmed by direct sequencing (Sangon Biotech, Shanghai, China).

### Statistical analysis

Statistical analysis was calculated by IBM SPSS statistics (version 26). Data are expressed as the mean ± standard deviation (SD) for the continuous variables and as a percentage of the total for categorical variables. The student’s t-test and ANOVA were used respectively to compare the means of two or three groups of continuous normally distributed variables. Inter-group differences in the single nucleotide polymorphism (SNP) genotype frequency were assessed using Pearson Chi-squared test or Fisher’s exact test. The possible risk genotype or allele towards the disease was assessed using binary logistic regression, to determine the odds ratios (OR) and 95% confidence intervals (95% CI). Moreover, the forest plots for the *FOXP3* rs2294021C/T and rs3761548C/A haplotypes were constructed based on calculations using logistic regression analysis, to observe the association between the combined genotype risk and disease. The genotype frequencies in the healthy controls were in accordance with the Hardy–Weinberg equilibrium (*p* > 0.05). A statistical value of *p* < 0.05 was considered significant.

## Results

### Demographic profile and clinical characteristics of the cases and controls

The demographic profiles and clinical characteristics of the patients and controls are summarized in Table 1. The HC group consisted of 200 healthy individuals, made up of 97 males (48.5%) and 103 females (51.5%). In the T2DM group, 115 (57.5%) patients were male and 85 (42.5%) were female, and in the T2DN group, 144 (72.0%) were male and 56 (28.0%) were female. The mean ages of the individuals in the HC, T2DM and T2DN groups were 57.57 ± 10.95, 61.64 ± 9.80 and 60.05 ± 10.82 years, respectively. The mean onset of diabetes age in the T2DM group was 48.48 ± 9.53 years, whereas in the in T2DN groups, it was 46.64 ± 9.96 years. The age at onset of T2DM, BMI, HDL cholesterol, urea, creatinine and urea/creatinine ratio showed significant variation between the T2DN and T2DM group (t-test, *p* < 0.05).

### Genotyping and sequencing analysis

**Fig. 1:**
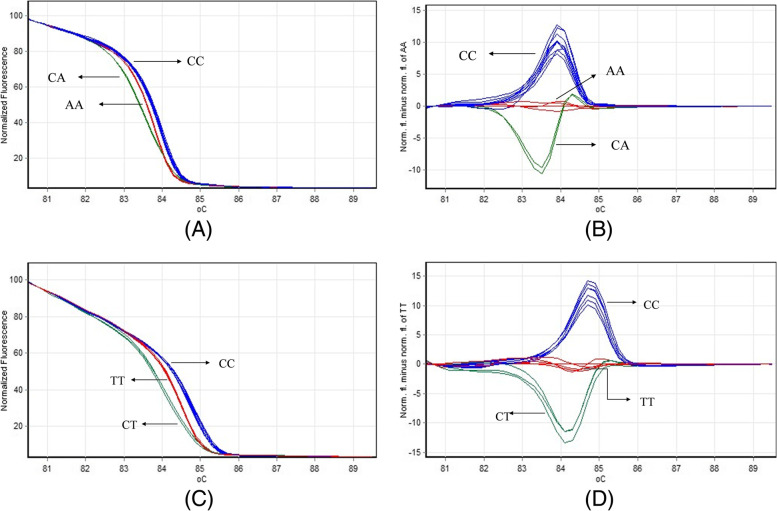
HRM assays for rs3761548C/A (**A**&**B**) and rs2294021C/T (**C**&**D**). **A**&**C** shows the normalized curve of fluorescence change in each small division of temperature change imparted onto the assay mix. The blue curves depict samples with wild-type (CC) while the red and green represents those with mutant genotype AA/TT and CA/CT respectively. **B**&**D** shows the normalized and temperature-shifted difference plot with three different melting profiles: the wild-type (CC) sample is blue, while the mutant homozygote (AA/TT) and heterozygote (CA/CT) are displayed red and green respectively

As shown in Fig. 1, normalized and temperature-shifted difference plots for rs3761548C/A and rs2294021C/T were constructed. Wild genotype, heterozygous and homozygous mutations could be accurately identified on the difference plot curves. The direct sequencing results confirmed the HRM analysis.

### Genotype distributions and allelic frequencies

The PCR-HRM results for the genotype and allelic distributions are illustrated in Table 2. For rs3761548C/A, the frequencies of the CC, CA and AA genotypes were respectively 67, 14 and 19% in the patients (72, 19, 9% in T2DM; 62.5, 9, 28.5% in T2DN) and 72.5, 18.5, 9% in the controls, which diverged significantly between HC and patients (χ2 = 10.452; *p* = 0.005), T2DM and T2DN (χ2 = 28.765; *p* < 0.001) groups. Furthermore, a significant correlation was noted between the allele frequencies of C and A (74 and 26% in the patients, 81.5 and 18.5% in the T2DM group, 67 and 33% in the T2DN group, 82 and 18% in the control group, respectively), with an OR of 0.625 (95% CI = 0.463–0.843; *p* = 0.002) for the HC vs. patient group and 0.461 (95% CI = 0.332–0.639; *p* < 0.001) for the T2DM vs. T2DN group. The wild type CC vs. other genotypes presented an OR of 0.530 (95% CI = 0.354–0.791; p = 0.002) and 0.648 (95% CI = 0.425–0.987; *p* = 0.043), and the homozygous AA vs. others genotype exhibited an OR of 4.897 (95% CI = 2.398–10.003; *p* < 0.001) and 4.030 (95% CI = 2.272–7.151; *p* < 0.001) in the HC vs. patients and T2DM vs. T2DN groups, respectively. For rs2294021, the genotype frequencies of CC, CT and TT were 41.5, 17 and 41.5% in the patients and 41, 27.5 and 31.5% in the control group, respectively, showing a significant difference between the HC and patients (χ2 = 10.672; *p* = 0.005), while there was no statistical difference in the allelic distribution. Both genotype and allelic frequencies differed significantly between the T2DM and T2DN group (*P* = 0.002 and P = 0.002, respectively). Further analysis demonstrated that participants with the heterozygous CT genotype (OR = 0.483; 95% CI = 0.281–0.831; *p* = 0.009) were protected, while those with the homozygous TT genotype were at higher risk of developing T2DN (OR = 1.946; 95% CI = 1.299–2.914; *p* = 0.001). Similar conclusions regarding the roles of the CT and TT genotypes were drawn in the HC vs. patients group, respectively (OR = 0.540, 95% CI = 0.360–0.816, *p* = 0.003; OR = 1.543, 95% CI = 1.078–2.208, *p* = 0.018).

**Table 2:** Genotype and allele frequency distribution of rs3761548C/A and rs2294021 Polymorphism among HC, T2DM and T2DN

		Genotype		χ2 (*p*-value)			Alleles	OR 95%CI	*p*-value	Group comparison	OR 95%CI	*p*-value
rs3761548		CC (%)	CA (%)	AA (%)		C	A					
	HC (200)	145 (72.5)	37 (18.5)	18 (9)	**10.452 (0.005)**	0.82	0.18	0.625 (0.463–0.843)	**0.002**	CC vs. Others	0.530 (0.354–0.791)	**0.002**
	Patients (400)	269 (67)	56 (14)	75 (19)		0.74	0.26			CA vs Others	0.855 (0.533–1.370)	0.514
										AA vs. Others	4.897 (2.398–10.003)	**< 0.001**
	T2DM (200)	144 (72)	38 (19)	18 (9)	**28.765 (< 0.001)**	0.815	0.185	0.461 (0.332–0.639)	**< 0.001**	CC vs. Others	0.648 (0.425–0.987)	**0.043**
	T2DN (200)	125 (62.5)	18 (9)	57 (28.5)		0.67	0.33			CA vs Others	0.422 (0.232–0.768)	**0.005**
										AA vs. Others	4.030 (2.272–7.151)	**< 0.001**
rs2294021		CC (%)	CT (%)	TT (%)		C	T					
	HC (200)	82 (41)	55 (27.5)	63 (31.5)	**10.672 (0.005)**	0.55	0.45	0.826 (0.650–1.051)	0.121	CC vs. Others	1.021 (0.723–1.441)	0.907
	Patients (400)	166 (41.5)	68 (17)	166 (41.5)		0.50	0.50			CT vs Others	0.540 (0.360–0.810)	**0.003**
										TT vs. Others	1.543 (1.078–2.208)	**0.018**
	T2DM (200)	89 (44.5)	44 (22)	67 (33.5)	**12.918 (0.002)**	0.55	0.45	0.643 (0.486–0.850)	**0.002**	CC vs. Others	0.781 (0.524–1.163)	0.224
	T2DN (200)	77 (38.5)	24 (12)	99 (49.5)		0.68	0.32			CT vs Others	0.483 (0.281–0.831)	**0.009**
										TT vs. Others	1.946 (1.299–2.914)	**0.001**

*OR* Odds Ratio and 95% CI, *HC* Healthy controls, *T2DM* Type 2 diabetes, *T2DN* Type 2 diabetic Nephropathy; patients = Female/Male (T2DN + T2DM)

### The gender-based evaluation

As the *FOXP3* gene is X-linked, three groups (T2DM, T2DN and HC) were stratified by sex and statistical analysis was performed. Additional gender-based data are presented in Table 3. At position rs3761548C/A in the females, the genotypes did not show significant differences between the female controls and patients, while the allele frequencies C and A (82 and 18% in the HC group, 73 and 27% in the patients, respectively) exhibited an OR of 0.593 (95% CI = 0.381–0.924; *p* = 0.021). A higher AA (14% vs. 4%) and lower CA (27% vs. 42%) was observed in the T2DN group compared to the T2DM group; this indicated a statistical significance in the genotype frequencies (χ2 = 9.503; *p* = 0.009), while being independent of the female allele frequency. ORs of 3.168 (95% CI = 1.027–9.777; *p* = 0.045) and 4.556 (95% CI = 1.153–17.997; *p* = 0.031) were observed for AA vs. others in female HC vs. patients and T2DM vs. T2DN respectively. Regarding rs3761548C/A in the male subjects, evaluations of the genotype distribution in the patients showed 73.0% for the CC genotype, 4.0% for the CA genotype, and 23.0% for the AA genotype. The corresponding data for the controls were 92.0% (CC), 3.0% (CA) and 5.0% (AA). Significant divergence in the genotype frequencies was observed between the HC and patients (χ2 = 15.923; *p* < 0.001), and between the T2DM and T2DN groups (χ2 = 15.944; *p* < 0.001162) groups. A higher percentage of male patients and T2DN patients were A carriers than male controls and T2DM patients respectively. Additionally, we noticed that CC vs. other genotypes had an OR of 0.238 (95% CI = 0.110–0.516; *p* < 0.001), AA vs. other genotypes had an OR of 5.428 (95% CI = 2.108–13.975; *p* < 0.001) between male controls and patients group; corresponding data between male T2DM and T2DN groups were 0.307 (95% CI = 0.166–0.569; *p* < 0.001176) and 3.439 (95% CI = 1.808–6.540; *p* < 0.001166), respectively.

**Table 3:** Gender-based data on genotype and allele frequency distribution of rs3761548C/A and rs2294021 Polymorphism among HC, T2DM and T2DN

			Genotype		χ2 (*p*-value)		Alleles	OR 95%CI	*p*-value	Group comparison	OR 95%CI	*p*-value
rs3761548		CC (%)	CA (%)	AA (%)		C	A					
Females	HC (103)	70 (68)	29 (28)	4 (4)	5.294 (0.071)	0.82	0.18	0.593 (0.381–0.924)	**0.021**	CC vs. Others	0.636 (0.374–1.083)	0.096
	Patients (141)	81 (58)	44 (31)	13 (23)		0.73	0.27			CA vs Others	1.157 (0.663–2.022)	0.607
										AA vs. Others	3.168 (1.027–9.777)	**0.045**
	T2DM (85)	46 (54)	36 (42)	3 (4)	**9.503 (0.009)**	0.75	0.25	0.857 (0.499–1.473)	0.577	CC vs. Others	1.216 (0.615–2.406)	0.574
	T2DN (56)	33 (59)	15 (27)	8 (14)		0.72	0.28			CA vs Others	0.371 (0.172–0.802)	**0.012**
										AA vs. Others	4.556 (1.153–17.997)	**0.031**
Males	HC (97)	89 (92)	3 (3)	5 (5)	**15.923 (< 0.001)**	0.93	0.07	0.214 (0.118–0.389)	**< 0.001**	CC vs. Others	0.238 (0.110–0.516)	**< 0.001**
	Patients (259)	188 (73)	12 (4)	59 (23)		0.75	0.25			CA vs Others	1.522 (0.420–5.515)	0.522
										AA vs. Others	5.428 (2.108–13.975)	**< 0.001**
	T2DM (115)	98 (85)	2 (2)	15 (13)	**15.944 (< 0.001162)**	0.86	0.14	0.299 (0.192–0.467)	**< 0.001**	CC vs. Others	0.307 (0.166–0.569)	**< 0.001176**
	T2DN (144)	92 (64)	3 (2)	49 (34)		0.65	0.35			CA vs Others	1.202 (0.197–7.318)	0.842
										AA vs. Others	3.439 (1.808–6.540)	**< 0.001166**
rs2294021		CC (%)	CT (%)	TT (%)		C	T					
Females	HC (103) Patients (141)	30 (29) 54 (38)	51 (50) 49 (35)	22 (21) 38 (27)	5.376 (0.068)	0.54 0.56	0.46 0.44	075 (0.749–1.542)	0.695	CC vs. Others	1.510 (0.877–2.602)	0.137
										CT vs Others	0.543 (0.323–0.913)	**0.021**
										TT vs. Others	1.358 (0.745–2.476)	0.317
	T2DM (85) T2DN (56)	36 (42)	27 (32)	22 (26)	1.559 (0.459)	0.58	0.42	0.770 (0.477–1.245)	0.770	CC vs. Others	0.645 (0.318–1.307)	0.224
		18 (32)	22 (39)	16 (29)		0.70	0.30			CT vs Others	1.390 (0.687–2.811)	0.360
										TT vs. Others	1.145 (0.538–2.440)	0.725
Males	HC (97) Patients (259)	52 (54) 112 (48)	4 (4) 19 (3)	41 (42) 128 (49)	3.533 (0.171)	0.58	0.42	0.647 (0.464–0.903)	**0.010**	CC vs. Others	0.659 (0.413–1.054)	0.082
						0.47	0.53			CT vs Others	1.841 (0.610–5.554)	0.279
										TT vs. Others	1.335 (0.833–2.137)	0.230
	T2DM (115) T2DN (144)	53 (46) 59 (41)	17 (15) 2 (1.4)	45 (39) 83 (57.6)	**20.454 (< 0.001036)**	0.86	0.14	0.621 (0.438–0.881)	**0.008**	CC vs. Others	0.812 (0.495–1.332)	0.409
						0.66	0.34			CT vs Others	0.081 (0.018–0.359)	**0.001**
										TT vs. Others	2.117 (1.284–3.488)	**0.003**

*OR* Odds Ratio and 95% CI, *HC* Healthy controls, *T2DM* Type 2 diabetes, *T2DN* Type 2 diabetic Nephropathy; patients = Female/Male (T2DN + T2DM)

At the rs2294021C/T position in females, neither genotypes nor alleles significantly differed between the controls and patients. In the male subjects, the frequencies of C and T were 58 and 42% in the control group, and 47 and 53% in the patients group, respectively; this indicated a statistical significance between the gene variants of this SNP in the controls and patients groups (*p* = 0.010), while no association was observed in the genotype frequencies (*p* = 0.171). Moreover, analysis of the genotype distribution (χ2 = 20.454; *p* < 0.001) and allele frequencies (OR = 0.621, 95% CI = 0.438–0.881; *p* = 0.008) suggested that there were significant correlations between the T2DM and T2DN group. We also noticed that the CT genotype possibly plays a protective role in the progression of T2DM toward T2DN (OR = 0.081; 95% CI = 0.018–0.359; *p* = 0.001), while the variant homozygote type had the opposite effect (OR = 2.117; 95% CI = 1.284–3.488; *p* = 0.003).

### The haplotype combination analysis

**Fig. 2:**
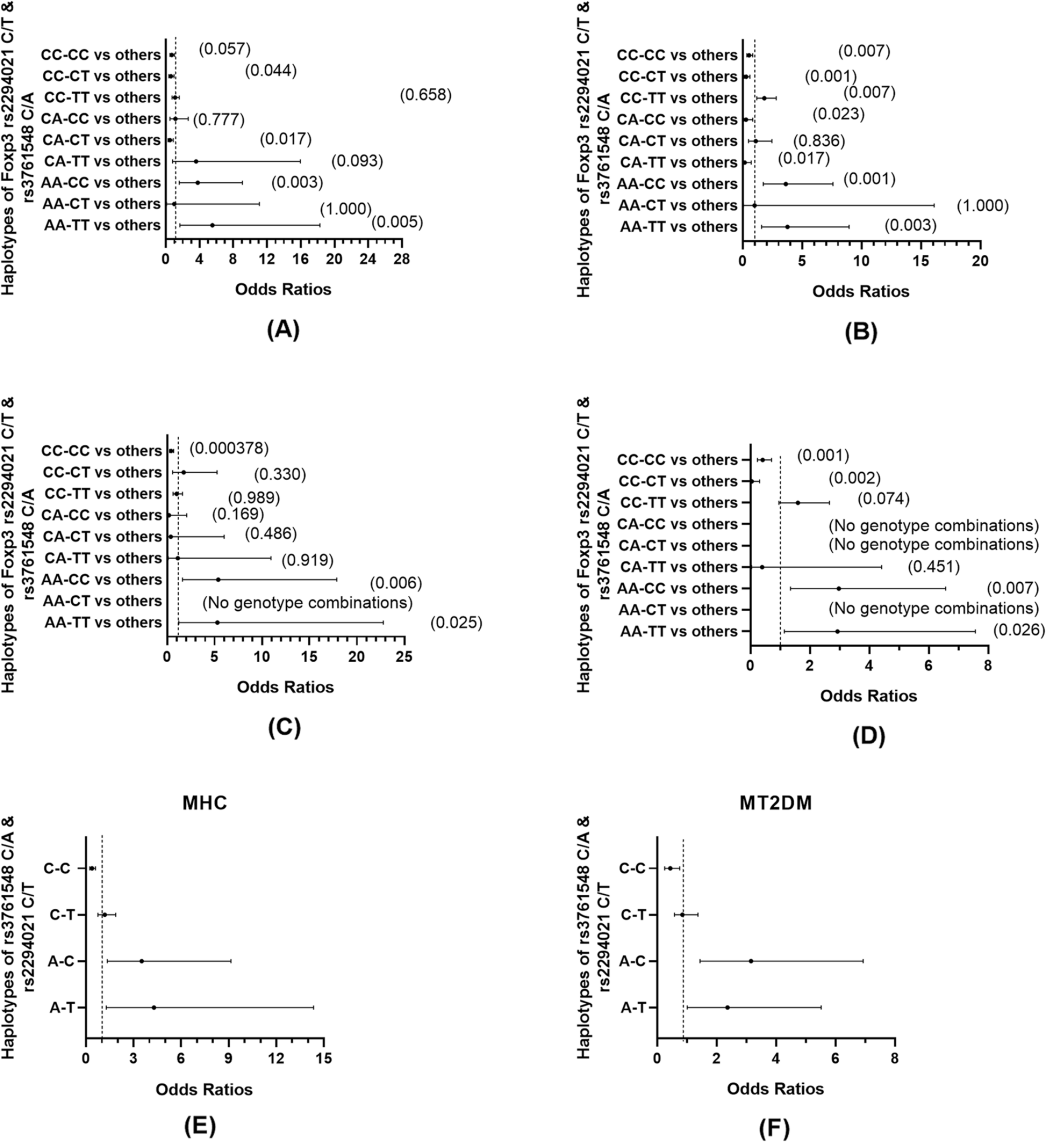
The distribution of genotype and allele combinations of rs3761548 C/A and rs2294021 C/T polymorphisms are displayed in the forest plot. **A** patients (T2DM + T2DN) vs. HC; **B** T2DM vs. T2DN (**C**) Male patients vs. Male HC (**D**) Male T2DM vs. Male T2DN (**E**) Male patients vs. Male HC (**F**) Male T2DM vs. Male T2DN

A comparison of the genotype combinations of the rs376548C/A and rs2294021C/T polymorphisms in the patients and control groups is shown in Fig. 2. AA-CC vs. others and AA-TT vs. others regarding genotype combinations had an OR of 3.793 (95% CI = 1.584–9.082; *p* = 0.003) and 5.517 (95% CI = 1.666–18.273; *p* = 0.005) in the HC vs. Patients group, and 3.619 (95% CI = 1.727–7.584; *p* = 0.001) and 3.760 (95% CI = 1.581–8.942; *p* = 0.003) in the T2DM vs. T2DN group, respectively, suggesting that AA-CC and AA-TT could be risk genotype combinations, whereas the CC-CT genotype combination (rs376548C/A-rs2294021C/T) was considered as a protective combination with an OR value of 0.600 (95% CI = 0.365–0.986; *p* = 0.044) in the HC vs. patients group and 0.287 (95% CI = 0.137–0.603; *p* = 0.001) in the T2DM vs. T2DN group.

Regarding males, the AA-CC vs. others and AA-TT vs. others groups displayed an OR of 2.970 (95% CI = 1.344–6.564; *p* = 0.007) and 2.930 (95% CI = 1.136–7.561; *p* = 0.026) in the HC vs. Patients group, 5.046(95% CI = 2.035–12.512; *p* = 0.000) and 3.093 (95% CI = 1.460–6.554; p = 0.003) in the T2DM vs. T2DN group respectively (Fig. 2A&B), while the CC-CC genotype combinations (rs376548C/A-rs2294021C/T) showed an OR value of 0.418 (95% CI = 0.258–0.676; *p* = 0.000378) in the male HC vs. Patients group. The CC-CC vs. others and CC-CT vs. others groups had OR value of 0.407 (95% CI = 0.234–0.708; p = 0.001) and 0.040 (95% CI = 0.005–0.308; *p* = 0.002) in the male T2DM vs. T2DN group, respectively (Fig. 2C&D). Further evaluations of the allele combinations in the C-C vs. others (OR = 0.378, 95% CI = 0.237–0.603; *p* = 0.00046), A-C vs. others (OR = 3.504, 95% CI =1.343–9.145; *p* = 0.010), and A-T vs. others (OR = 4.280, 95% CI = 1.277–14.344; *p* = 0.018) groups between the male T2DM and T2DN group were also conducted (Fig. 2E&F). Regarding the females, neither statistically significant differences in the combined genotypes nor any differences in allele combinations among female individuals were observed.

## Discussion

As a typical chronic inflammatory disorder, T2DM is caused by the interactions between multiple self-reactive cells and Treg subpopulations, which may contribute to organ and/or tissue damage. FOXP3-expressing Tregs are indispensable for metabolic fitness and regulatory function, as *FOXP3* is an essential transcriptional modulator and metabolic gatekeeper for the down-regulation of an effective immune response [13]. The protective role of anti-inflammatory Tregs has been demonstrated in animal models [11]. In diabetic (db/db) mice, antibody-mediated depletion of Tregs aggravated insulin resistance and exacerbated diabetic nephropathy, whereas, adoptive transfer of Tregs significantly improved insulin sensitivity and diabetic nephropathy by tipping the balance towards anti-inflammation and inhibiting CD8+ effector cell infiltration into the visceral adipose tissue (VAT) and kidneys. Therefore, we hypothesized that the observed variations in the transcription factor *FOXP3* gene may affect the risk of T2DM and T2DN by indirectly altering the function of *FOXP3* and/or its expression. In the present study, the impact of *FOXP3* polymorphisms (rs3761548C/A and rs2294021C/T) on T2DM and T2DN in the Han Chinese population was evaluated in detail, as well as from a gender dimorphism perspective.

rs3761548C/A is located between CNS2 and CNS3, which can bind to transcription factors (TFs) and play a crucial role in the regulation of *FOXP3* gene [28]. Consistent with earlier studies that dealt with T2DM and T2DN susceptibility among the South Indian population, our finding showed that the subjects with an AA mutant genotype had 5-fold and 4-fold increased risk of T2DM and T2DN susceptibility respectively, while the CA genotype had a protective effect against diabetic nephropathy in the Chinese population. Besides, this polymorphism also increased the risk of UC and was related to a lower expressions of *FOXP3* in the colonic tissues of the studied population [39]. Zheng et al. also reported that rs3761548 mutation not only resulted in an increased risk of graves (GD) but also contributed to reduced relative luciferase activity of the *FOXP3* promoter as well as the decreased mRNA expression of *FOXP3* in patients with GD [44]. In summary, the presence of risk allele A may be associated with the occurrence and progression of immune-related diseases. A functional report of this polymorphism also supported our findings, they found that carriers of the A allele abolished binding to the E47 and c-Myb transcription factors, leading to the defective transcription of the *FOXP3* gene, and the AA genotype was also shown to be associated with a decreased expression of *FOXP3* compared to other genotypes [6]. To be more specific, the rs3761548 SNP, located in the core of the “GGGCGG” sequences, may potentially alter the expression of the *FOXP3* gene by affecting the interaction between the transcription factors and the *FOXP3* promoter, such as specificity protein-1 (sp-1), which directly binds to DNA through specific motifs and enhances gene transcription [20]. Mantel et al. identified that the *FOXP3* promoter is located 6.2 kb upstream of the *FOXP3* translational start site, and further study revealed that several basal transcriptional elements are situated in the core promoter, such as TATA, GC and CAAT boxes [25]. In addition to this, the T cell receptor (TCR)–calcium–calcineurin signaling pathway has been reported to be a cornerstone of the adaptive immune response and is critical for the activation, differentiation and regulation of Tregs. A panel of transcription factors, including the TCR-induced nuclear factor of activated T cells (NFAT) proteins accompanied by activator protein-1 (AP-1), binds to the *FOXP3* promoter and participates in *FOXP3* regulation [37]. In addition, the Sp-1- and IL-2-induced STAT5 bind to the promoter of *FOXP3* gene and positively regulates its expression [5, 29]. Several GATA-3 binding sites within the *FOXP3* promoter negatively regulate *FOXP3* expression, indicating that IL-4-induced Th2 cell differentiation overrules Treg differentiation [25, 29]. It is reasonable to speculate that the *FOXP3* promoter rs3761548 SNP, located in the upstream region, may indirectly interfere with the calcium–calcineurin signaling pathway, enhancing immune activation and inflammation, as well as the initiation and progression of T2DM and T2DN. On the other hand, a published study showed that *Runx1* binds to RORγt and that *FOXP3* exerts positive and negative effects on the IL-17 transcription, respectively [42]. Accordingly, this genetic polymorphism of the *FOXP3* promoter region might interfere with the formation of a transcription complex that breaks the balance between regulatory and effector cells, resulting in a significant decrease in CD4^+^CD25^+^FOXP3^+^ Treg cell levels, and an increase in Th1 and Th17 cells levels in the periphery of patients with T2DN from an earlier study [41].

Another concerned polymorphism was rs2294021C/T. It was identified that TT was likely to be a risk genotype affecting the predisposition to T2DM and T2DN, which could influence the *FOXP3* expression levels and the number of Treg cells, and resulting in a pro-inflammatory environment to some extent. Besides, it is necessary to point out that rs2294021 is located in the coiled-coil domain-containing protein 22 (CCDC22), which has been uncovered in the context of X-linked intellectual disability (XLID) and implicated it can play an essential role in the activation of NF-κB (a master regulator of the inflammatory and innate immune responses by regulating the genes that encode inflammatory cytokines, such as tumor necrosis factor-α (TNF-α), interleukin-1β (IL-1β) and interleukin-6 (IL-6)) [21]. NF-κB has been demonstrated to be a marker of progressive T2DN and exerts an impact on the inflammatory pathway. Moreover, previous studies have shed light on renal inflammation stimulated by the activation of the NF-κB pathway, which triggered insulin resistance and enhanced renal gluconeogenesis in T2DM [24]. Therefore, this polymorphism could be responsible for the stability, localization, and translation efficiency of the *CCDC22* gene, accordingly, leading to the abnormal activation of NF-κB, which may affect the inflammatory pathways. Besides, rs2294021 polymorphism is also relevant to the function of *FOXP3,* since its position is in the complementary strand and in close proximity to *FOXP3* [14, 33]. Xia et al. also reported that rs2294021(C/T) may indirectly affect the transcription of *FOXP3* mRNA by forming linkage disequilibrium (LD) with other functional loci of *FOXP3*, such as rs3761548(C/A) [39].

Apart from those above, the joint effect of the combined genotypes of the two polymorphisms was described. AA-CC and AA-TT constitute significant risk factors, whereas CC-CT and CC-TT act as converse factors for both T2DM and T2DN. Therefore, it was hypothesized that the polymorphism at the promoter site (rs3761548C/A) appeared to be more determinant, regardless of the genotype at the intron site (rs2294021C/T). The results of this study were also confirmed in the males. One of the reasons for this hypothesis may be attributed to the fact that the promoter is involved in initiating transcription and interacts with many important cis-acting elements that regulate gene expression. Therefore, promoter regions may influence *FOXP3* expression by altering the binding specificity of the transcription factors to their binding sites and changing the dynamics of transcription initiation. Thus, the promoter region may have polymorphisms that are more closely related to function, potentially reflecting the *FOXP3* expression level by altering the binding specificity of transcription factors to their binding sites and by modifying the kinetics of transcription initiation [15].

Subsequently, a significant difference based on gender stratification was observed, especially for the rs2294021(C/T) SNP. We found that TT was associated with an approximately 2-fold higher hazard for the progression of nephropathy in males, while there was no noticeable association in females. Notably, these findings are further supported by Tipton et al., who reported that the number of Tregs in male rat kidneys was lower than that in females [36]. It can therefore be speculated that the mutant genotype TT is associated with lower numbers of Treg cells with lower expression of *FOXP3* and weaker suppressive function, which makes male subjects more inclined to develop chronic inflammatory disorders. Additionally, male-female differences in the sex chromosomal complement, XX vs. XY, have been studied. The genes encoded on the sex chromosomes or the skewing of X-chromosomal activation may result in sex differences in immune regulation and the development of immune-related disorders [23]. A previous study reported that genes on the alternative X chromosome can interact with the *FOXP3* locus through their influence on X inactivation, thus affecting the expression of *FOXP3* [3]. It has extensively been shown in the literature that males and females differ in their susceptibility to diseases involving immune responses. And the reason for these differences is multifactorial, probably induced by the complex interactions between the sex hormones, genes and environment, the progression of DN is no exception [1]. Numerous studies have indicated that the mechanisms involved in the initiation and progression of kidney inflammation are sex-dependent [35]. Males with diabetes have been reported to have a higher risk of DN than females with diabetes [1, 26]. Female sex hormones, such as endogenous E2, which regulates pro-inflammatory responses transcriptionally mediated by NF-κB, are thought to be protective against DN [18, 26]. Although sex differences are known to be involved in the processes contributing to DN, their precise mechanisms are far from being understood. More prospective and randomized clinical studies will reveal male–female differences, help better understand the progression of DN in both sexes and define novel targets for the more effective prevention and treatment of the condition using personalized medicine.

In summary, our findings be inconsistent with their gender-based evaluations from India. Such discrepancies between our two studies may mainly be due to the ethnic/racial background of the subjects, which may depend on the genotype or allele frequency, genome location on chromosomes, different LD patterns in the populations, and various environmental or social factors. Secondly, the inclusion/exclusion criteria for sample selection established by different experimental groups and genotyping methodologies should also be considered. Besides, Abdukassimova et al. mentioned a thought-provoking view that epigenetics may mediate the interactions between genes and the environment by regulating the gene expression without changing the original DNA sequence, and thus could be responsible for the associations of *FOXP3* variants by engaging specific potential biological pathways/genes across different racial/ethnic populations [2, 12]. Despite this, characterization and functional studies of polymorphisms of the *FOXP3* gene are required to explore and elucidate their pathogenic roles. More studies relating to *FOXP3* SNPs in larger cohort prospective studies among different ethnicities, will contribute to clarifying their mechanism in T2DM and T2DN.

## Conclusions

In conclusion, this study provides the first evidence of a genetic association between rs3761548C/A and rs2294021C/T and susceptibility to diabetes and diabetic nephropathy, as well as the first sex-stratified evaluation in the Han Chinese population, particularly suggesting the implication of sex dimorphism in the progression of male diabetic nephropathy. An additional combined effect analysis of the genotypes also indicated the possible involvement of the two SNPs in diabetes and diabetic nephropathy. To gain further insight into the role of inflammation-associated X-linked polymorphisms in the genetic susceptibility to T2DM and T2DN, larger-sample studies and following cell and animal model-based studies regarding the function of *FOXP3* polymorphisms are required.

## Data Availability

The datasets used and/or analysed during the current study are available from the corresponding author on reasonable request.
